# SPECTRUM: early clinical experience from the first global real-world study of aflibercept 8 mg in patients with neovascular age-related macular degeneration

**DOI:** 10.1038/s41433-026-04260-3

**Published:** 2026-01-30

**Authors:** Clare Bailey, Clemens Lange, Varun Chaudhary, Paolo Lanzetta, Hassiba Oubraham, Martin Kirchner, Tobias Machewitz, Helmut Allmeier, Xin Zhang, Zoran Hasanbasic, Marion R. Munk

**Affiliations:** 1 Department of Ophthalmology, University Hospitals Bristol and Weston NHS Foundation Trust, Bristol, UK; 2 Eye Center, Faculty of Medicine, Albert-Ludwig University Freiburg, Freiburg, Germany; 3 Department of Ophthalmology, St. Franziskus Hospital, Münster, Germany; 4 Department of Surgery, McMaster University, Hamilton, ON Canada; 5 Department of Medicine–Ophthalmology, University of Udine, Udine, Italy; 6 Istituto Europeo di Microchirurgia Oculare (IEMO), Udine, Italy; 7 Centre OPHTA-45, Montargis, France; 8 Bayer AG, Leverkusen, Germany; 9 Bayer AG, Berlin, Germany; 10 Bayer Consumer Care AG, Basel, Switzerland; 11 Augenarzt Praxisgemeinschaft Gutblick AG, Pfäffikon, Switzerland; 12 Department of Ophthalmology, University Hospital Bern, Bern, Switzerland; 13 Northwestern University, Feinberg School of Medicine, Chicago, IL USA

**Keywords:** Macular degeneration, Outcomes research

## Introduction

The CANDELA and PULSAR trials demonstrated the efficacy and safety of intravitreal aflibercept 8 mg in the treatment of neovascular age-related macular degeneration (nAMD) [1, 2]. SPECTRUM is the first global real-world study of aflibercept 8 mg in nAMD, and its unique study design enables rolling global and country cohort analyses. Here, we describe early clinical experience in the first ~100 patients with treatment-naïve (TN) or previously treated (PT) nAMD to have a visit and visual acuity (VA) assessment at Week 8 (W8) in SPECTRUM.

## Methods

SPECTRUM (NCT06075147) is a 24-month, prospective observational study (February 2024–September 2027) being conducted across 18 countries among patients with TN or PT nAMD aged ≥50 years who have been prescribed aflibercept 8 mg by their attending physician. All treatment decisions are made by each patient’s physician in accordance with local clinical practice. Enrollment criteria are reported in the ClinicalTrials.gov record [3]. The study protocol was approved by each study site’s independent ethics committee/institutional review board. All participants provided written informed consent. The W8 analysis described here was prespecified; all data were analysed descriptively.

## Results

**Fig. 1:**
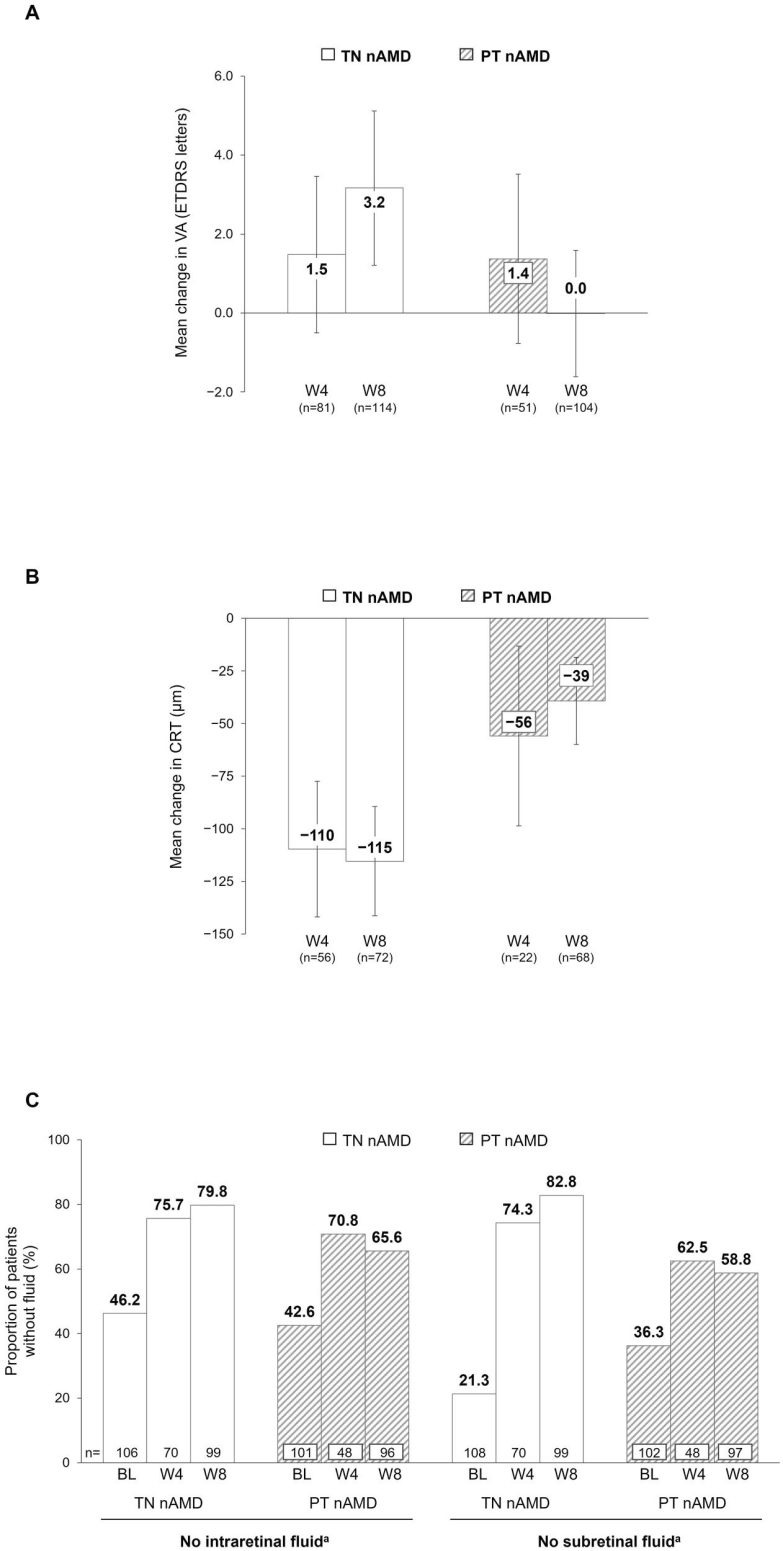
Functional and anatomic outcomes in patients with nAMD at Week 8 in SPECTRUM. **A** Mean change in VA from baseline at Week 8. **B** Mean change in CRT from baseline at Week 8. **C** Proportion of patients without intraretinal fluid or subretinal fluid at Week 8. Data are for the FAS (observed cases); error bars represent 95% CI. Mean VA/CRT change at Week 4 and Week 8 from baseline was calculated in patients with a VA/CRT assessment at Week 4 and Week 8, respectively. Week 4 = visits closest to 28 (14–42) days after the first injection (BL), and Week 8 = visits closest to 56 (43–70) days after BL. Note that outcomes described here would not reflect the effect of an injection received at Week 8, and some patients may not have received an injection at this timepoint. *BL* baseline, *CRT* central retinal thickness, *ETDRS* Early Treatment Diabetic Retinopathy Study, *FAS* full analysis set (all patients receiving ≥1 dose of study drug plus ≥1 post-baseline assessment), *nAMD* neovascular age-related macular degeneration, *PT* previously treated, *TN* treatment-naïve, *VA* visual acuity, *W* week. ^a^Fluid data were collected per investigator discretion; the presence of intraretinal fluid and subretinal fluid was determined by optical coherence tomography with the instrument available at each study site, and the proportions presented here were calculated based on the number of patients who had an assessment at each of the indicated timepoints.

Baseline characteristics are shown in Table 1. Patients received a mean ± SD (median) of 3.0 ± 0.3 (3) and 2.6 ± 0.6 (3) injections until Day 70 after baseline in the TN and PT nAMD cohorts, respectively (first injection received at baseline). In the TN and PT nAMD cohorts, mean (95% CI) change in VA from baseline at W8 was +3.2 (1.2, 5.1) and 0.0 (−1.6, 1.6) letters, respectively (Fig. 1A), and mean change in central retinal thickness was −115 (−141, −89) and −39 (−60, −19) µm, respectively (Fig. 1B). The proportions of patients without intraretinal fluid or subretinal fluid increased from baseline to W8 in both cohorts (Fig. 1C). Note that the W8 injection data include any injections received at W8, whereas the W8 outcomes reflect the injections administered before the W8 visit.

**Table 1:** Baseline demographics and disease characteristics of patients in the SPECTRUM Week 8 analysis of the treatment-naïve and previously treated nAMD cohorts.

	Treatment-naïve nAMD (*N* = 114)	Previously treated nAMD (*N* = 104)
Age, years	80.8 ± 7.1	79.5 ± 7.3
Female, *n* (%)	69 (60.5)	60 (57.7)
Race, *n* (%)^a^		
Asian	8 (7.0)	0
White	75 (65.8)	90 (86.5)
Not reported	31 (27.2)	14 (13.5)
MNV type, *n* (%)		
Type 1	34 (29.8)	29 (27.9)
Type 2	19 (16.7)	11 (10.6)
Mixed^b^	0	0
Type 3	6 (5.3)	5 (4.8)
Missing/unknown/not applicable	55 (48.2)	59 (56.7)
Visual acuity, ETDRS letters^c^	60.1 ± 17.4	61.6 ± 19.4
Central retinal thickness, µm^d^	358 ± 110	316 ± 102
Median time (range) since nAMD diagnosis, months	0.2 (0.0, 21.9)	36.9 (1.4, 178.9)
Prior treatment for nAMD, *n* (%)		
Aflibercept 2 mg	–	56 (53.9)
Faricimab 6 mg	–	18 (17.3)
Ranibizumab 0.5 mg	–	15 (14.4)
Bevacizumab (variable)	–	4 (3.9)
Brolucizumab 6 mg	–	3 (2.9)
Other	–	1 (1.0)
Steroid	–	0
Missing	–	7 (6.7)

FAS. Data are mean ± SD unless otherwise stated; percentages may not add up to 100 due to rounding. *ETDRS* Early Treatment of Diabetic Retinopathy Study, *FAS* full analysis set (all patients receiving ≥1 dose of study drug plus ≥1 post-baseline assessment), *MNV* macular neovascularisation, *nAMD* neovascular age-related macular degeneration.

^a^Data on race were collected in Australia, Canada, Germany, Italy, Japan, Portugal, South Korea, Saudi Arabia, Spain, Switzerland, United Arab Emirates and the United Kingdom only; for France, Denmark, Finland, Netherlands, Norway and Sweden, data on race were not collected according to local law/regulations.

^b^Combination of MNV Type 1 and Type 2.

^c^Visual outcomes were assessed during routine clinical practice and reported in ETDRS letter scores; where ETDRS charts were unavailable, approximate Snellen scores were converted to ETDRS letter scores.

^d^Central retinal thickness was determined based on physician discretion using optical coherence tomography with the instrument available at each study site.

In the TN and PT nAMD cohorts, ocular treatment-emergent adverse events (TEAEs) in the study eye occurred in 3/114 (2.6%) and 4/104 (3.9%) patients, respectively, and non-ocular TEAEs were observed in 5/114 (4.4%) and 0 patients, respectively. Two ocular TEAEs in the PT nAMD cohort were considered study drug–related (increased intraocular pressure and vitreous floaters; *n* = 1 each). There were no serious ocular or non-ocular TEAEs in either cohort, nor any cases of intraocular inflammation.

## Discussion

This prespecified W8 analysis of the SPECTRUM study provides valuable insights into early treatment responses to aflibercept 8 mg in diverse real-world settings among clinically heterogenous patients with nAMD. Patients received their first injection at baseline; by the W8 visit, the TN nAMD cohort reported VA gains, and both the TN and PT nAMD cohorts reported improvements in CRT and absence of fluid. The safety profile was consistent with that reported for the PULSAR trial [1].

SPECTRUM is an observational study, and analyses are exploratory only. Findings from this W8 analysis are based on ~100 patients only in each cohort, and this patient subset may not be fully reflective of the overall global SPECTRUM nAMD cohorts.

## Supplementary information

SPECTRUM study investigators

## Data Availability

Availability of the data underlying this publication will be determined later according to Bayer’s commitment to the European Federation of Pharmaceutical Industries and Associations/Pharmaceutical Research and Manufacturers of America (EFPIA/PhRMA) ‘Principles for responsible clinical trial data sharing.’ This pertains to the scope, time point, and process of data access. As such, Bayer commits to sharing upon request from qualified scientific and medical researchers participant-level clinical trial data, study-level clinical trial data, and protocols from clinical trials in participants for medicines and indications approved in the United States (US) and European Union (EU) as necessary for conducting legitimate research. This applies to data on new medicines and indications that have been approved by the EU and US regulatory agencies on or after 1 January 2014. Interested researchers can use www.clinicalstudydatarequest.com to request access to anonymised participant-level data and supporting documents from clinical studies to conduct further research that can help to advance medical science or improve patient care. Information on the Bayer criteria for listing studies and other relevant information is provided in the study sponsor’s section of the portal. Data access will be granted to anonymised participant-level data, protocols, and clinical study reports after approval by an independent scientific review panel. Bayer is not involved in the decisions made by the independent review panel. Bayer will take all necessary measures to ensure that participant privacy is safeguarded.
