# Supplementary material for: SPECTRUM: early clinical experience from the first global real-world study of aflibercept 8 mg in patients with neovascular age-related macular degeneration
Source: Eye (Lond). 2026 Jan 30;40(5):735–8. doi: 10.1038/s41433-026-04260-3 (PMC13013631; doi:10.1038/s41433-026-04260-3)
Supplement: Supplementary file 1 — SPECTRUM study investigators [file 41433_2026_4260_MOESM1_ESM.pdf]

SPECTRUM Study Investigators

Study Site Name	City	Country	Full Name	Study Role
Retina and Macula Specialists Miranda	Sydney	Australia	Adrian Fung	Primary Investigator
Specialist Eye Group	Melbourne	Australia	Amy Cohn	Primary Investigator
Sydney Retina Clinic and Day Surgery	Sydney	Australia	Andrew Chang	Primary Investigator
Vision Eye Institute Boronia	Melbourne	Australia	Devinder Chauhan	Primary Investigator
South West Retina	Liverpool	Australia	Gerald Liew	Primary Investigator
Marsden Eye Institute Parramatta	Parramatta	Australia	Jennifer Arnold	Primary Investigator
Sydney West Retina	Sydney	Australia	Paul Mitchell	Primary Investigator
Retina Specialists Victoria Warragul	Warragul	Australia	Sanjeewa Wickremasinghe	Primary Investigator
Samad Opthalmology	Halifax	Canada	Arif Samad	Primary Investigator
UHN	Toronto	Canada	Brian Ballios	Primary Investigator
Retine de l'est	Montreal	Canada	Cynthia Qian	Primary Investigator
Eye Health MD	Montreal	Canada	David Lederer	Primary Investigator
Trimed Eye Centre	Orillia	Canada	Donald Nixon	Primary Investigator
Toronto Retina	Toronto	Canada	Keyvan Koushan	Primary Investigator

SPECTRUM Study Investigators

VRMTO	Toronto	Canada	Netan Choudhry	Primary Investigator
Mississauga Retina Institute	Mississauga	Canada	Parnian Arjmand	Primary Investigator
Retina Centre of Ottawa	Ottawa	Canada	Raman Tuli	Primary Investigator
Retina Surgical Associates	New Westminster	Canada	Robert Gizicki	Primary Investigator
Uptown Eye Specialists	Vaughan	Canada	Sohel Somani	Primary Investigator
SJHC London Eye	London	Canada	Thomas Sheidow	Primary Investigator
RSJH	Hamilton	Canada	Varun Chaudhary	Primary Investigator
Vancouver General Hospital	Vancouver	Canada	Wai Ching Lam	Primary Investigator
Aalborg University Hospital - Eye department	Aalborg	Denmark	Lasse Jørgensen Cehofski	Primary Investigator
Rigshospitalet Glostrup- Afdeling for Øjensygdomme	Glostrup	Denmark	Michael Larsen	Primary Investigator
Hospital of Southern Jutland -Department of Eye Diseases	Esbjerg	Denmark	Michael Spangsberg	Primary Investigator
Tampere University hospital	Tampere	Finland	Hannele Uusitalo-Järvinen	Primary Investigator
Helsinki Retina Research Group	Helsinki	Finland	Raimo Tuuminen	Primary Investigator
Institut d'ophtalmologie Sourdille Atlantique	Saint-Herblain	France	Alexandre Bourhis	Primary Investigator
Hôpital Lariboisière Fernand-Widal	Paris	France	Aude Couturier	Primary Investigator

SPECTRUM Study Investigators

Hôpital Avicenne	Bobigny	France	Audrey Giocanti-Auregan	Primary Investigator
Centre Ophtalmologique Maison rouge	Strasbourg	France	Benjamin Wolff	Primary Investigator
Hôpital Edouard Herriot HCL	Lyon	France	Corinne Dot	Primary Investigator
Paris Retina Vision	Paris	France	David Sayag	Primary Investigator
CHIC Créteil	Créteil	France	Eric SOUIED	Primary Investigator
Pole Vision Val d'Ouest	Lyon	France	Flore De Bats	Primary Investigator
Institut Ophtalmologique de l'Ouest	Nantes	France	Frank Becquet	Primary Investigator
Centre Monticelli Paradis	Marseille	France	Frédéric Matonti	Primary Investigator
Ophta 45	Loiret	France	Hassiba Oubraham-Mebroukine	Primary Investigator
Centre Ophtalmologique Saint Exupéry	Lyon	France	Hessam Razavi	Primary Investigator
Centre Ophtalmologie des Arceaux	Montpellier	France	Isabelle Aubry-Quenet	Primary Investigator
Clinique Mathilde	Rouen	France	Joël Uzzan	Primary Investigator
Centre d'Exploration de la rétine Kléber	Lyon	France	Laurent Berthon	Primary Investigator
Hôpital de la Croix Rousse	Lyon	France	Laurent Kodjikian	Primary Investigator
Centre Rétine Gallien	Bordeaux	France	Laurent Velasque	Primary Investigator

SPECTRUM Study Investigators

CHU de Bordeaux	Bordeaux	France	Marie-Noelle Delyfer	Primary Investigator
Centre Opthalmologie Sorbonne Saint Michel	Paris	France	Mathieu Lehmann	Primary Investigator
Centre d'Ophtalmologie du Dauphiné	Grenoble	France	Mathilde Gallice	Primary Investigator
Centre Aix Vision	Aix en Provence	France	Mélanie Tessier	Primary Investigator
Centre d'Exploration de l'Odeon	Paris	France	Olivia Zambrowski	Primary Investigator
Clinique des yeux	Bordeaux	France	Philippe Schauer	Primary Investigator
CHU François Mitterrand Dijon	Dijon	France	Pierre-henry Gabrielle	Primary Investigator
Centre Ophtalmologique d'Imagerie et de Laser	Paris	France	Salomon Cohen	Primary Investigator
CHU Pasteur	Nice	France	Stéphanie Baillif	Primary Investigator
CHU amiens	Amiens	France	Thi Ha Chau Tran	Primary Investigator
Clinique Honoré Cave	Montauban	France	Vincent Gualino	Primary Investigator
Clinique de l'Union	Saint-Jean	France	Xavier Benouaich	Primary Investigator
MVZ AR Augenblick Rheinland GmbH	Brühl	Germany	Alexander Daudrich	Primary Investigator
Uniklinikum Würzburg, Augenklinik	Würzburg	Germany	Andreas Berlin	Primary Investigator
Universitätsmedizin Greifswald, Klinik und Poliklinik für Augenheilkunde	Greifswald	Germany	Andreas Stahl	Primary Investigator

SPECTRUM Study Investigators

Augenzentrum Berliner Ring	Würzburg	Germany	Astrid Sader-Moritz	Primary Investigator
Augenklinik am Wittenbergplatz	Berlin	Germany	Ben Mehrinfar	Primary Investigator
Diakonie Klinikum Dietrich Bonhoeffer	Neubrandenburg	Germany	Christian Karl Brinkmann	Primary Investigator
Augenklinik Universitätsklinikum Heidelberg	Heidelberg	Germany	Christoph Arnholdt	Primary Investigator
Augenzentrum am St. Franziskus-Hospital	Münster	Germany	Clemens Lange	Primary Investigator
Dr. med. Costin Mihaescu	Würzburg	Germany	Costin-Ilie Mihaescu	Primary Investigator
Visualeins GmbH MVZ für Augenheilkunde	Osnabrück	Germany	Erik Beeke	Primary Investigator
Marienhospital Osnabrück	Osnabrück	Germany	Fabian Höhn	Primary Investigator
St. Elisabeth-Krankenhaus GmbH	Katharinenberg	Germany	Hüsnü Berk	Primary Investigator
Rund ums Auge GbR	Grefrath	Germany	Ines Lanzl	Primary Investigator
Sankt-Gertrauden-Krankenhaus GmbH Abteilung Augenheilkunde	Berlin	Germany	Joachim Wachtlin	Primary Investigator
Augenklinik Petrisberg (Medical eye research xperts Institut GmbH)	Trier	Germany	Kai Januschowski	Primary Investigator
Augenklinik und Poliklinik, Universitätsmedizin Mainz	Mainz	Germany	Katrin Lorenz	Primary Investigator
ASKLEPIOS Klinik Nord	Hamburg	Germany	Marc Schargus	Primary Investigator
Universitätsklinikum Regensburg	Regensburg	Germany	Maria-Andreéa Gamulescu	Primary Investigator

SPECTRUM Study Investigators

Smile Eyes Augenärzte, MVZ Augenärzte am Airport	Frankfurt am Main	Germany	Nikolaus Feucht	Primary Investigator
Charité Berlin, Campus Benjamin Franklin, Klinik für Augenheilkunde	Berlin	Germany	Oliver Zeitz	Primary Investigator
Makula Center Augsburg, Südblick Augenzentren	Augsburg	Germany	Philipp Müller	Primary Investigator
Augenzentrum im Medizeum	Mainz	Germany	Ralf Schmitt	Primary Investigator
BeyondEye GmbH	Bonn	Germany	Roxana Fulga	Primary Investigator
Universitätsklinikum Aachen, Augenheilkunde	Aachen	Germany	Sabine Baumgarten	Primary Investigator
Kadina Research Zschopau	Zschopau	Germany	Simo Murovski	Primary Investigator
Stiftung Pius- Hospital Universitätsklinik für Augenheilkunde	Oldenburg	Germany	Stefan Schrader	Primary Investigator
Augenzentrum Frankfurt	Frankfurt am Main	Germany	Svenja Deuchler	Primary Investigator
Universitätsklinikum Köln, Augenheilkunde	Cologne	Germany	Tim U. Krohne	Primary Investigator
MVZ Augenheilkunde Mitteldeutschland GmbH	Leipzig	Germany	Tobias Duncker	Primary Investigator
Universitätsklinikum Tübingen, Department für Augenheilkunde	Tübingen	Germany	Tobias Peters	Primary Investigator
Gemeinschaftspraxis für Augenheilkunde	Munich	Germany	Ulrich Thelen	Primary Investigator
MVZ der Klinik Dardenne GmbH Makulazentrum	Bonn	Germany	Vassiliki Romanou-Papadopoulou	Primary Investigator
Klinikum Chemnitz, Augenheilkunde	Chemnitz	Germany	Vinodh Kakkassery	Primary Investigator

SPECTRUM Study Investigators

Philipps-Universität Marburg, Klinik für Augenheilkunde	Marburg	Germany	Walter Sekundo	Primary Investigator
Miulli - Acquaviva delle Fonti	Acquaviva delle Fonti	Italy	Alfonso Savastano	Primary Investigator
Ospedale Maggiore di Trieste	Trieste	Italy	Daniele Tognetto	Primary Investigator
Ospedale Molinette - Torino	Turin	Italy	Enrico Borrelli	Primary Investigator
Policlinico Tor Vergata - Roma	Rome	Italy	Federico Ricci	Primary Investigator
Ospedale Monaldi - Napoli	Naples	Italy	Flavia Chiosi	Primary Investigator
S. Raffaele - Milano	Milan	Italy	Francesco Maria Bandello	Primary Investigator
Ospedale Maggiore Policlinico di Milano	Milan	Italy	Francesco Viola	Primary Investigator
Ospedale Sacco di Milano	Milan	Italy	Giovanni Staurenghi	Primary Investigator
Ospedale SS Annunziata - Chieti	Chieti	Italy	Leonardo Mastropasqua	Primary Investigator
Ospedale Parini - Aosta	Aosta	Italy	Luca Ventre	Primary Investigator
Ospedale Umberto I di Ancona	Ancona	Italy	Marco Lupidi	Primary Investigator
Policlinico "Paolo Giaccone" - Palermo	Palermo	Italy	Maria Vadalà	Primary Investigator
Presidio Gaspare Rodolico - Catania	Catania	Italy	Niccolò Castellino	Primary Investigator
Ospedale S. Maria della Misericordia-Udine	Udine	Italy	Paolo Lanzetta	Primary Investigator

SPECTRUM Study Investigators

Ospedale San Martino - Genova	Genoa	Italy	Raffaella Rosa	Primary Investigator
Aoyagi Eye Clinic	Tokyo	Japan	Aoyagi Koji	Primary Investigator
Osaka University Hospital	Osaka	Japan	Chikako Hara	Primary Investigator
Fujimura Arimatsu Eye Clinic	Nagoya	Japan	Fujimura Shigeto	Primary Investigator
Fujiwara Eye Clinic	Kyoto	Japan	Fujiwara Shintaro	Primary Investigator
Muramatsu Eye Clinic	Hamamatsu	Japan	Hagiwara Akira	Primary Investigator
Otsuka Eye Clinic	Tokyo	Japan	Handa Sou	Primary Investigator
Chukyo Eye Clinic	Nagoya	Japan	Ichikawa Reiko	Primary Investigator
Chukyo Hospital	Nagoya	Japan	Kaga Tatsushi	Primary Investigator
Kaneda Eye Clinic	Osaka	Japan	Kaneda Masahiro	Primary Investigator
Yokkaichi Municipal Hospital	Yokkaichi	Japan	Kawano Kenichi	Primary Investigator
Nagata Eye Clinic	Fukuoka	Japan	Kimura Hideya	Primary Investigator
Kimura Eye Hospital	Hiroshima	Japan	Kimura Yugo	Primary Investigator
Kozawa Eye Hospital and Diabetes Center	Gifu	Japan	Kishino Genichiro	Primary Investigator
Miyake Eye Hospital	Nagoya	Japan	Kondo Nagako	Primary Investigator

SPECTRUM Study Investigators

Sannoudai Hospital Ganka Naika Clinic	Yokohama	Japan	Kuribara Isao	Primary Investigator
Yamagata University Hospital	Yamagata	Japan	Masahiko Sugimoto	Primary Investigator
Fuchu Eye Center	Fuchu	Japan	Mishima Soichiro	Primary Investigator
Hirota Eye Clinic	Osaka	Japan	Miyagi Hidetaka	Primary Investigator
Kitano Hospital	Osaka	Japan	Miyahara Shinsuke	Primary Investigator
Iida Municipal Hospital	Iida	Japan	Mori Toshio	Primary Investigator
Kimitsu Chuo Hospital	Kimitsu	Japan	Nakamura Yosuke	Primary Investigator
Mikawa Eye Clinic	Okazaki	Japan	Nishimura Tomohisa	Primary Investigator
Nagoya Medical Center	Nagoya	Japan	Okado Satoshi	Primary Investigator
Sato Ganka Clinic	Sendai	Japan	Sato Hiroaki	Primary Investigator
Kobe University Hospital	Kobe	Japan	Sentaro Kusuhara	Primary Investigator
Sugita Eye Hospital	Kumamoto	Japan	Sugita Iichiro	Primary Investigator
Takagi Ophthalmic Hospital	Nagoya	Japan	Takagi Mariko	Primary Investigator
Takasu Eye Clinic	Toyohashi	Japan	Takasu Ippei	Primary Investigator
Nakatake Eye Clinic	Fukuoka	Japan	Umeda Naoyasu	Primary Investigator

SPECTRUM Study Investigators

Yamane Eye Clinic	Matsue	Japan	Yamane Ken	Primary Investigator
Omiya Nanasato Eye Institute	Saitama	Japan	Yamazaki Kenichiro	Primary Investigator
Sapporo Kato Ophthalmology Clinic	Sapporo	Japan	Yuji Kato	Primary Investigator
UMCG	Groningen	Netherlands	Angela Huiskamp	Primary Investigator
Erasmus MC	Rotterdam	Netherlands	Hans Vingerling	Primary Investigator
ETZ	Tilburg	Netherlands	Janneke Van Lith-Verhoeven	Primary Investigator
Jeroen Bosch Ziekenhuis	’s-Hertogenbosch	Netherlands	Marianne Smeets	Primary Investigator
Maastricht UMC	Maastricht	Netherlands	Sankha Amarakoon	Primary Investigator
Radboud UMC	Nijmegen	Netherlands	Yara Lechanteur	Primary Investigator
LUMC	Leiden	Netherlands	Yvonne de Jong-Hesse	Primary Investigator
Drammen Sykehus, Øyeavdelingen, Vestre Viken HF	Drammen	Norway	Marius Dalby	Primary Investigator
Sykehuset Østfold, Moss -Eye department	Moss	Norway	Tayyab Ahmad	Primary Investigator
UNIDADE LOCAL DE SAÚDE DA REGIÃO DE LEIRIA E.P.E.	Leiria	Portugal	António Campos	Primary Investigator
ALM SERVIÇOS OFTALMOLOGIA MÉDICA E CIRÚRGICA, S.A.	Lisbon	Portugal	Carlos Marques Neves	Primary Investigator
Hospital Garcia de Orta - ULS de Almada Seixal, EPE	Almada	Portugal	Diogo Cabral	Primary Investigator

SPECTRUM Study Investigators

Instituto de Retina de Lisboa	Lisbon	Portugal	João Nascimento	Primary Investigator
UOC | Unidade de Oftalmologia de Coimbra SA	Coimbra	Portugal	João Pedro Marques	Primary Investigator
Instituto de Microcirurgia Ocular (IMO)	Barcelona	Portugal	José Roque	Primary Investigator
Unidade Local de Saúde Lisboa Ocidental - Hospital de Egas Moniz	Lisbon	Portugal	Maria Picoto	Primary Investigator
MAGRABI Hospitals & Centers	Jeddah	Saudi Arabia	Ahmed Ibrahim	Primary Investigator
King Khaled Eye Specialist Hospital	Riyadh	Saudi Arabia	Hassan Al-Dhibi	Primary Investigator
MAGRABI Hospitals & Centers	Jeddah	Saudi Arabia	Hussein Elgendy	Primary Investigator
MAGRABI Hospitals & Centers	Jeddah	Saudi Arabia	Maher Almalis	Primary Investigator
King Khalid University Hospital	Riyadh	Saudi Arabia	Marwan Abouammoh	Primary Investigator
Severance Hospital	Seoul	South Korea	Christopher SeungKyu Lee	Primary Investigator
Hangil Eye Hospital	Incheon	South Korea	Daniel Duck-Jin Hwang	Primary Investigator
Samsung Medical Center	Seoul	South Korea	Don-Il Ham	Primary Investigator
Kim's Eye Hospital	Seoul	South Korea	HanJoo Cho	Primary Investigator
Pusan national University Hospital	Busan	South Korea	Iksoo Byon	Primary Investigator
Seoul National University Hospital	Seoul	South Korea	Kyu Hyung Park	Primary Investigator

SPECTRUM Study Investigators

YeungNam University Hospital	Daegu	South Korea	Min Sagong	Primary Investigator
Seoul National University Bundang Hospital	Seongnam	South Korea	Se Joon Woo	Primary Investigator
ChungNam National University Hospital	Daejeon	South Korea	Young-Joon Jo	Primary Investigator
H MERIDA	Mérida	Spain	Alvaro Galván Gallego	Primary Investigator
H Leon	León	Spain	Amancia Mateos Hernández	Primary Investigator
H San Pedro	Logroño	Spain	Ana Isabel Oca Lázaro	Primary Investigator
H Terrassa	Terrassa	Spain	Bárbara Delás Alós	Primary Investigator
H Donostia	San Sebastián	Spain	Cristina Irigoyen Laborra	Primary Investigator
HU NAVARRA	Pamplona	Spain	Daniel Aliseda Pérez de Madrid	Primary Investigator
H SON LLATZER	Palma de Mallorca	Spain	Elena Rigo Oliver	Primary Investigator
HU GALDAKAO	Galdakao	Spain	Erika Vázquez Cruchaga	Primary Investigator
H VIRGEN MACARENA	Seville	Spain	Estanislao Gutiérrez Sánchez	Primary Investigator
CHU IMI GRAN CANARIA	Las Palmas	Spain	Francisco Antonio Cabrera López	Primary Investigator
HCU LOZANO BLESA	Zaragoza	Spain	Francisco Javier Ascaso Puyuelo	Primary Investigator
H CIVIL MALAGA	Málaga	Spain	Gustavo Fernández-Baca Vaca	Primary Investigator

SPECTRUM Study Investigators

H CLINIC BARCELONA	Barcelona	Spain	Javier Zarranz Ventura	Primary Investigator
HU BELLVITGE	L'Hospitalet de Llobregat	Spain	Luis Arias Barquet	Primary Investigator
H Caceres	Cáceres	Spain	María Concepción Rodríguez Villace	Primary Investigator
H SON ESPASES	Palma de Mallorca	Spain	María Victoria Gómez Resa	Primary Investigator
H NAVAL FERROL	Ferrol	Spain	Nuria Olivier Pascual	Primary Investigator
HOSPITAL MIGUEL SERVET	Zaragoza	Spain	Óscar Ruiz Moreno	Primary Investigator
HU BURGOS	Burgos	Spain	Renzo Renato Portilla Blanco	Primary Investigator
H Esperança	Barcelona	Spain	Vladimir Poposki Hamamdjieva	Primary Investigator
Ögonmottagning Falu Lasarett	Falun	Sweden	Aseel Modher Raghib	Primary Investigator
Ögonläkarna i Eslöv AB	Eslöv	Sweden	Ingar Bergstrand	Primary Investigator
Sunderby Sjukhus, Ögonkliniken	Luleå	Sweden	Inger Westborg	Primary Investigator
Skaane University Hospital Lund - Eye clinic A	Lund	Sweden	Marion Silvia Schroeder	Primary Investigator
Uppsala University hospital - Eye clinic	Uppsala	Sweden	Yazgülü Yumusak	Primary Investigator
Swiss Visio Retina Research Center / Swiss Visio Montchoisi	Lausanne	Switzerland	Aude Ambresin	Primary Investigator
Hôpital Ophtalmique Jules-Gonin	Lausanne	Switzerland	Chiara Eandi	Primary Investigator

SPECTRUM Study Investigators

Kantonsspital St. Gallen, Augenklinik (KSSG)	St. Gallen	Switzerland	Christophe Valmaggia	Primary Investigator
Universitätsspital Zürich, Augenklinik USZ	Zurich	Switzerland	Daniel Barthelmes	Primary Investigator
Stadtspital Zürich Triemli, Augenklinik	Zurich	Switzerland	Gábor Somfai	Primary Investigator
Berner Augenklinik (Swiss Eye Institute)	Bern	Switzerland	Justus Garweg	Primary Investigator
Vista Augenklinik, CH-4102 Binningen	Binningen	Switzerland	Katja Hatz	Primary Investigator
Augenarzt Praxisgemeinschaft Gutblick AG: Bern, Aarau, Wetzikon, Wallisellen	Bern	Switzerland	Marion Munk	Primary Investigator
LUKS Augenklinik	Lucerne	Switzerland	Martin Schmid	Primary Investigator
Universitätsspital Bern, Klinik für Augenheilkunde	Bern	Switzerland	Martin Zinkernagel	Primary Investigator
Clinica di Oftalmologa (INSI), Ospedale Regionale di Lugano (EOC)	Lugano	Switzerland	Moreno Menghini	Primary Investigator
Imperial College London- Al Ain Branch	Al Ain	United Arab Emirates	Alaa Attawan	Primary Investigator
Moorfields Eye Hopsital	Dubai	United Arab Emirates	Ammar Safar	Primary Investigator
Magrabi Eye Hospital - DHCC	Dubai	United Arab Emirates	Amr Farid	Primary Investigator
Cleveland Clinic Abu-Dhabi	Abu Dhabi	United Arab Emirates	Aniruddha Agarawal	Primary Investigator
Kent and Canterbury Hospital	Canterbury	United Kingdom	Afsar Jafree	Primary Investigator
Bedford Hospital	Bedford	United Kingdom	Aires Lobo	Primary Investigator

SPECTRUM Study Investigators

York Hospital	York	United Kingdom	Archana Airody	Primary Investigator
Worcestershire Royal Hospital	Worcester	United Kingdom	Chandoshi Mukherjee	Primary Investigator
Bristol Eye Hospital	Bristol	United Kingdom	Clare Bailey	Primary Investigator
Gloucestershire Royal Hospital	Gloucester	United Kingdom	Emily Fletcher	Primary Investigator
Southampton	Southampton	United Kingdom	Gabriella De Salvo	Primary Investigator
Royal Victoria Infirmary	Newcastle upon Tyne	United Kingdom	Hani Hasan	Primary Investigator
Liverpool University Hospital	Liverpool	United Kingdom	Ian Pearce	Primary Investigator
Hull University Teaching Hospitals NHS Trust	Hull	United Kingdom	Louise Downey	Primary Investigator
Frimley Health NHS Foundation Trust	Frimley	United Kingdom	Manju Chandran	Primary Investigator
Sunderland Eye Infirmary	Sunderland	United Kingdom	Michael Grinton	Primary Investigator
St. James's University Hospital	Leeds	United Kingdom	Narendra Dhingra	Primary Investigator
Wolverhampton Eye Infirmary	Wolverhampton	United Kingdom	Niro Narendran	Primary Investigator
Western Eye Hospital	London	United Kingdom	Saad Younis	Primary Investigator
University Hospitals of Leicester	Leicester	United Kingdom	Vasileios Konidaris	Primary Investigator
